# Block Compressive Sensing (BCS) Based Low Complexity, Energy Efficient Visual Sensor Platform with Joint Multi-Phase Decoder (JMD)

**DOI:** 10.3390/s19102309

**Published:** 2019-05-19

**Authors:** Mansoor Ebrahim, Wai Chong Chia, Syed Hasan Adil, Kamran Raza

**Affiliations:** 1 Faculty of Engineering, Sciences and Technology, Iqra University, Karachi 75500, Pakistan; hasan.adil@iqra.edu.pk (S.H.A.); kraza@iqra.edu.pk (K.R.); 2 Faculty of Sciences and Technology, Sunway University, Bandar Sunway 47500, Malaysia; waichongc@sunway.edu.my

**Keywords:** visual sensor networks, multi-camera nodes, image processing, compressive sensing, computational complexity, low-cost image sensors, practical model, image reconstruction, joint multi-phase decoding (JMD)

## Abstract

Devices in a visual sensor network (VSN) are mostly powered by batteries, and in such a network, energy consumption and bandwidth utilization are the most critical issues that need to be taken into consideration. The most suitable solution to such issues is to compress the captured visual data before transmission takes place. Compressive sensing (CS) has emerged as an efficient sampling mechanism for VSN. CS reduces the total amount of data to be processed such that it recreates the signal by using only fewer sampling values than that of the Nyquist rate. However, there are few open issues related to the reconstruction quality and practical implementation of CS. The current studies of CS are more concentrated on hypothetical characteristics with simulated results, rather than on the understanding the potential issues in the practical implementation of CS and its computational validation. In this paper, a low power, low cost, visual sensor platform is developed using an Arduino Due microcontroller board, XBee transmitter, and uCAM-II camera. Block compressive sensing (BCS) is implemented on the developed platform to validate the characteristics of compressive sensing in a real-world scenario. The reconstruction is performed by using the joint multi-phase decoding (JMD) framework. To the best of our knowledge, no such practical implementation using off the shelf components has yet been conducted for CS.

## 1. Introduction

A visual sensor network (VSN) [1] is a wireless platform consists of a set of visual nodes, intermediate nodes, and a gateway. Visual nodes are the end devices responsible for capturing and sending the visual information to the intermediate nodes and are usually powered by batteries [2]. The information will then be relayed to a workstation via a gateway. As the use of VSN is increasing rapidly (i.e., visual nodes are getting smaller, and networks are growing larger), a set of new challenges in terms of energy consumption and bandwidth utilization is of significant consideration. One of the solutions to improve these issues is to trade the encoder (CPU) performance for reducing the amount of data transmission by using compression [3]. Research suggests that compressive sensing (CS) [4], an emerging technique, has the potential to serve as an efficient compression method for a visual sensor network (VSN), due to the simple-encoder complex-decoder paradigm, which is the inverse of traditional compression. On the one hand, the visual nodes, which serve as the encoders, are only required to quantize and transmit the measurements produced by CS. On the other hand, the server, which acts as a joint decoder, will perform the complex task of exploiting the correlations and redundancies of information collected by different visual nodes. It reduces the amount of processing to be done on the encoder, resulting in better energy consumption and bandwidth utilization

However, there are a few challenges in using CS for compression that includes reconstructing the images from a minimal sample set of data and practical implementation of CS at the encoder. To the best of our knowledge, the current studies of CS are more concentrated towards hypothetical characteristics with simulated results, rather than on the understanding the potential issues in the practical implementation of CS and its validation. Also, no practical evaluation of the reconstruction quality and computational complexity (energy consumption, memory utilization, and execution time) of CS is available. Furthermore, there is a lack of visual node prototypes that are capable of taking raw images (uncompressed imaged); most of the embedded cameras only provide output in JPEG format. As this research work is focused towards the implementation and analysis of compression technique BCS on VSN, capturing of images in raw format is important. This is because, in raw format, an image is not compressed by the camera and contains the complete information of the image while, in most of the standard formats—such as jpeg, png, MPEG, etc.—the output image is already in the form of compressed bit stream at capturing level resulting in some loss of data. Unless the bit stream is uncompressed on the spot to reconstruct the image, it is hard to directly apply other processing onto the compressed bitstream. 

Although there are existing VSN platforms [5,6,7,8,9,10,11,12,13,14,15]—such as Cyclops, MeshEyes, Citric, WiCa, SeedEyes, Eye-RIS, Panoptes, CMUcam4, CMUcam5/PIXY, iMote2/IMB400, and ArduCam to name a few—most of them are based on ad-hoc development of the visual part and do not have an efficient image compression implemented on the visual node. For example, CMUCam4 is capable of capturing a raw image; the capturing process is performed on a row-by-row basis. It means that if the observed scene consists of a moving object, then a row of image data might seem unconnected to the next row of image data. Another prototype such as MeshEyes is bulk in size and Cyclops can only capture raw images at low resolution. Therefore, it is necessary to develop a new visual node prototype that is capable of capturing raw images. 

In this research, a practical visual sensor platform is developed with efficient BCS mechanism using an Arduino Due microcontroller board, XBee transmitter, and uCAM-II camera. In this case, images or videos captured by the camera are first compressed using block compressive sensing [16] (BCS) on the microcontroller. The measurements produced from the compression are then transmitted from the encoder to the server via XBee transmitter. At the server, the received data is first decoded by BCS to recover the images. The images then go through the joint multi-phase decoding (JMD) [17] framework, where the correlations among the images are exploited. The exploited information is used to improve the visual quality of the decoded images. The JMD involves three main steps, namely (i) image registration, (ii) image fusion, and (iii) residual compensation. Depending on the deployment and configuration of the visual nodes, the entire compression scheme can handle the changes with minimum recalibration or reprogramming. Overall, the scheme can cope with three setups, (i) multi-view image, (ii) single-view video, and (iii) multi-view video. In addition, the developed visual node is also capable of capturing images and recording videos. However, this research work is limited to multi-view image scenario, and BCS along with JMD framework [17,18,19,20] is implemented on low power applications (VSN) to evaluate and analyze its performance based on the reconstruction quality and computational complexity of BCS rather than measuring the bandwidth utilization. 

The rest of the paper is organized as follows. The literature related to the application of CS in WSN is discussed in Section 2. Section 3 describes the hardware and software components used to construct the VSN platform. This is followed by the experimental setup in Section 4, and the evaluation results are presented and discussed. Finally, the paper is concluded in Section 5.

## 2. Literature Review

In this section, the review of different applications of CS for sensor networks data gathering and energy efficiency have been conducted [21,22,23,24,25,26,27]. In [21], the analysis of the application of CS on WSN is conducted. The analysis presented in the paper indicates that CS provides promising developments to reduce the specific constraints of WSN (power utilization, lifetime, time delay, cost) and validates its effectiveness in WSN. The CS scheme merges the data gathering and compression into a single step. Therefore, transmitting the whole image, only a smaller volume of measurements is necessary to be transmitted or stored. This paper helps in identifying the developments shown by the application of CS in WSN. 

The performance (energy, latency) analysis of CS for data gathering in WSN is carried out in [22]. Firstly, the paper highlights the issues related to data gathering in WSN and then proposes a few solutions, i.e., tree-based and gossip-based protocols scalable with energy and latency necessities. The simulation results of the proposed protocols show improved performance for data gathering in WSN in terms of energy and latency. Though, a tree-based protocol is exposed to the link lost. 

Conversely, [23] presents the first comprehensive design for CS to collect data for large scale WSN. The proposed model will help reduce the communication cost without increasing computational complexity, improve lifetime, and can handle uncommon sensor outputs competently. Furthermore, the proposed scheme is tested practically, and the results validate its capability and robustness. Though, the scheme is not suitable for small-scale sensor networks (partial signal sparsity). 

The work of [24] examines the improvements that can be attained by CS for data gathering in WSN. Two different methods were proposed, i.e., plan-CS and hybrid-CS based on a specific data gathering mechanism. The schemes were articulated and solve flow-based optimization problems. Yet, the simulation results indicate that plan-CS approach does not show any progress, while, the hybrid CS approach shows considerable improvement that can be observed in the throughput. Furthermore, the results were only verified for low-power applications. 

A detailed review and analysis of the state of the art CS schemes are presented in [25]. The survey was carried in two steps. The first step caters the security aspect of CS based on various random measurements matrices Gaussian matrix, circulate matrix, and other special random matrices that are the basis for applications in secure wireless communications. In the second step, the applications of secure CS based on various communication scenarios are reviewed. 

In [26], a review of various efficient wireless processes and enabling techniques to identify different varieties of correlated processes is conducted. This will help in the implementation of CS-based processing techniques to efficiently encounter limited radio resources and network infrastructure. Furthermore, different state-of-the-art CS schemes are analyzed, and a variety of promising new ideas relevant to large-scale antenna arrays, non-orthogonal multiple accesses (NOMA), and ultra-dense network (UDN) solutions are discussed and analyzed.

A complete assessment of different distributed coding schemes used to encode images in VSN is conducted in [27]. The research work includes the introduction of each algorithm along with its benefits and deficits when employed in VSN. The algorithms are then compared based on the defined criteria to determine which of the algorithm is suitable for VSN. The analyses show that CS provides better when compared with other schemes based on the defined criteria and is an appropriate solution for VSN energy and bandwidth issues.

## 3. Proposed BCS Visual Sensor Platform

As discussed earlier, there exist various VSN platforms. However, most of them do not have an efficient compression implemented on the visual node. In this regard, a prototype is developed to implement and evaluate the BCS scheme.

### 3.1. Overview

**Figure 1:**
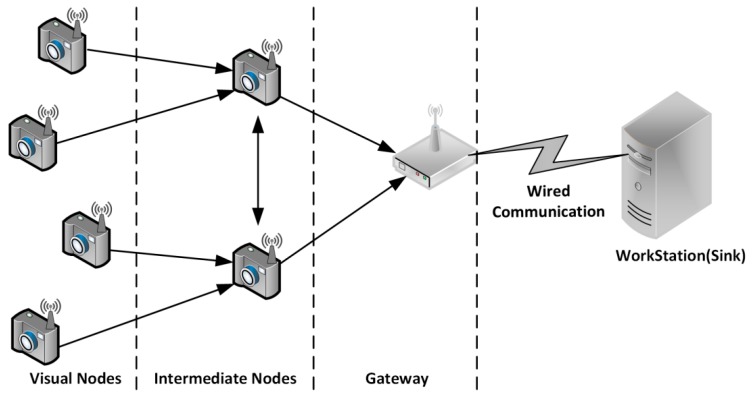
Architecture of a VSN.

A VSN platform primarily consists of hardware and software components. The hardware component includes the camera, processing unit, and transmission module that work together to create a visual node that is capable of capturing and sending the data to the workstation for further processing. Whereas the software component includes image acquisition, encoding process, and communication protocol that helps to compress and packetize the data before transmission. As shown in Figure 1 is an example of how devices in VSN are typically connected. The development of the platform also aims to create a simple, flexible, and low-cost VSN platform integrated with energy efficient compression. 

The main motivations in designing the proposed platform are:

- To have an off-the-shelf solution that is easily reproducible using existing low cost and widely available hardware components. We create a visual sensor by combining Arduino board, with an external uCAM-II camera and XBee transmission module. The uCAM-II camera is used to capture image data that will be processed and compressed on the Arduino board before they are transmitted via the XBee transmission module.
- To implement BCS on the visual sensor to reduce the amount of data that needs to be processed and transmitted. BCS is adapted to create a simple-encoder/complex-decoder paradigm that is preferable for VSN. It shifted most of the complex computation to the server and helped to prolong the lifetime of the devices that are powered by batteries.
- To implement and evaluate the JMD framework for the reconstruction of images using real-world data.

Details of the hardware and software components used to implement the proposed scheme are provided in the following subsections.

#### 3.1.1. Hardware Components

**Figure 2:**
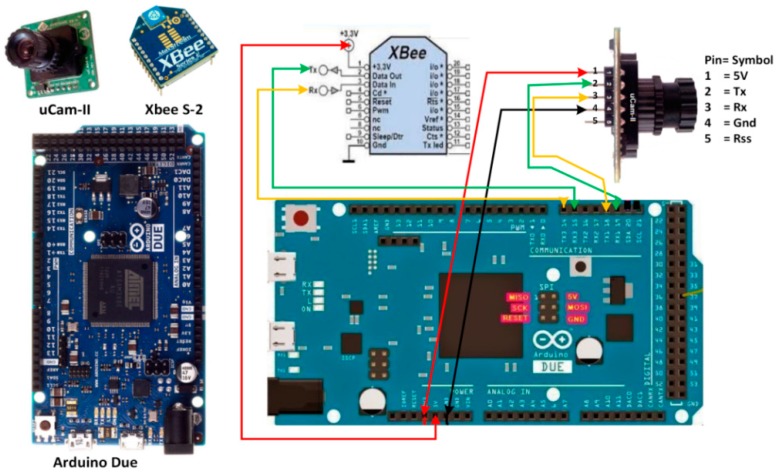
Standalone visual node built using Arduino Due, uCAM-II, and XBee [31].

As shown in Figure 2, a visual node that consists of an Arduino Due board [28], a CMOS uCAM-II camera [29], and an XBee transmission module [30].

1. Arduino Due Board

Although there are several other microcontrollers available, Arduino is a low-cost card-size board that offers sufficient processing power and memory for simple computation tasks. Moreover, its functionalities can be extended by connecting to many other peripherals (or shields), the code developed for one model can be reprogrammed and run on other Arduino board with minimum modifications. In the development of the proposed BCS visual node, an Arduino Due board [28] is selected. It is equipped with an Atmel SAM3X8E ARM Cortex-M3 microcontroller running at 84 MHz, 96 KB of SRAM memory, and 512 KB of flash memory. In addition to this, it also comes with several UART interfaces that can be used to communicate with other external components. The reason of selecting Due over other Arduino boards is that it uses less energy (runs at 3.3 V), higher computing performance (clock speed of 84 MHz), and has more SRAM and flash memory. Overall, it is difficult to implement the image processing task on other Arduino boards due to the limited amount of memory.

2. uCAM-II CMOS Camera

Among the many low-power, low-cost CMOS cameras [32,33,34,35,36], the uCAM-II by 4D schemes is selected [29] for the development of the BCS visual node. Unlike the other available cameras that only provide images in JPEG format, the uCAM-II is capable of providing images in both raw and jpeg formats. Furthermore, uCAM-II can capture images at resolution ranges from 80 × 60 to 640 × 480. Moreover, the uCAM-II is also compatible with lenses of different viewing angles. These include the standard 56-degree lens that comes together with uCAM-II, as well as the 76-degree lens and the 116-degree lens can be purchased as additional components. It operates on normal 5V DC supply, and no external DRAM is required for storing the images. The uCAM-II is connected to the Arduino Due board through one of the UART interfaces at 115,200 bauds.

3. XBee Wireless Module

Wireless communication between the visual node and the server is performed by using an XBee module. It can send and receive data via the 2.4 GHz or 900 MHz band at relatively low power. They can be used to set up a simple point-to-point link by using the transparent mode or to form a complex self-healing network that spread over a large area when using the API mode [37]. For the development of the BCS visual node, the XBee module is configured to operate in the API mode. In this case, the visual data is enclosed in a packet before transmission takes place. The XBee module is connected to the Arduino Due board through another UART interface. However, 125,000 bauds are used because the communication between the XBee module and the Due board is not reliable at 115,200 bauds given the Due’s clock frequency of 84 MHz [37].

#### 3.1.2. Software Components

**Figure 3:**
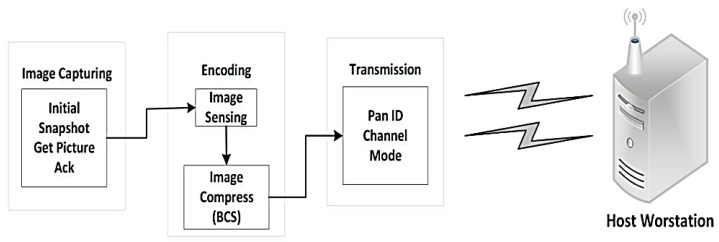
Software components associated with the visual node.

In this context, the software architecture is built using modular design. As shown in Figure 3, the platform consists of data preprocessing in the sensor side, control protocol during the transmission, and stream management in the server. We will summarize several key components in the rest of this section.

1. Image Capture

In our implementation, we capture an 8-bit grayscale raw image and store the image data in the Arduino flash memory for further processing. As Arduino Due has a larger flash memory than SRAM, it is better first to store the large image data into flash memory using PRGMEM variable modifier and then read the data from flash memory back into SRAM using a block-by-block approach.

**Figure 4:**
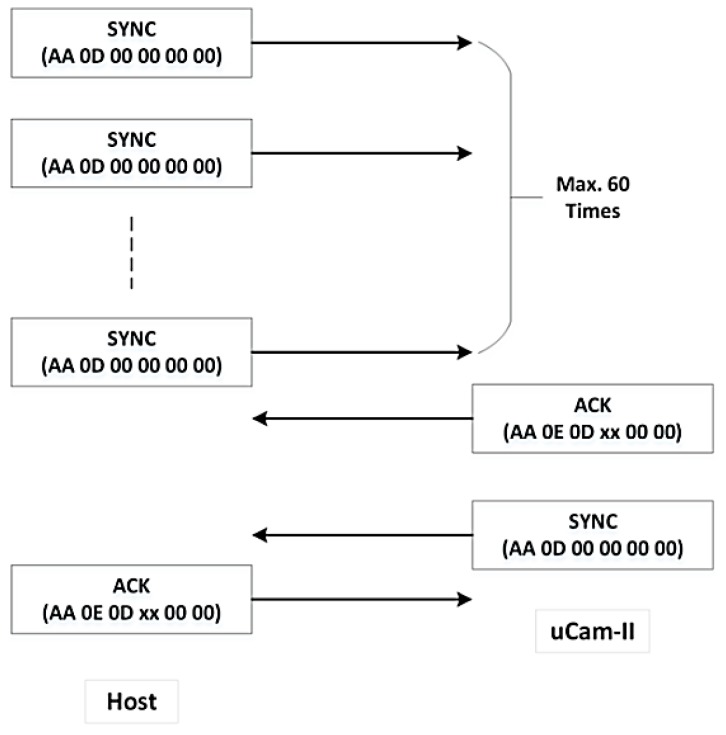
Synchronization process between uCAM-II and host.

To start the communication process, a connection between the host and the uCAM-II must be established. As shown in Figure 4, this is started by synchronizing the host with the uCAM-II via SYNC command. The SYNC command is sent periodically to awake the camera from sleep state if no commands have been sent. If communications are occurring between the host and the camera, the camera will stay awake. The host sends the SYNC command continuously until an acknowledgment (ACK) and SYNC command is received from the uCAM-II. A maximum of 60 SYNC command can be sent to awake the module. If the module does not respond after 60 SYNC commands, it is restarted, and the same actions are performed again. Usually, up to 25 to 60 SYNC commands may be necessary before the module will respond. After the host receives the response, it should reply with the ACK command to confirm the synchronization process.

After the communication link is established, uCAM-II is ready to capture images. In order to capture a raw image, the following commands have to be sent from the host to the uCAM-II.

- **INITIAL** is first used to configure the image size and image format.
- **SNAPSHOT** is to instruct uCAM-II to capture an image and store it in the buffer.
- **GET PICTURE** is used to request an image from the uCAM-II.
- **ACK** is sent to indicate the end of the last operation.

**Figure 5:**
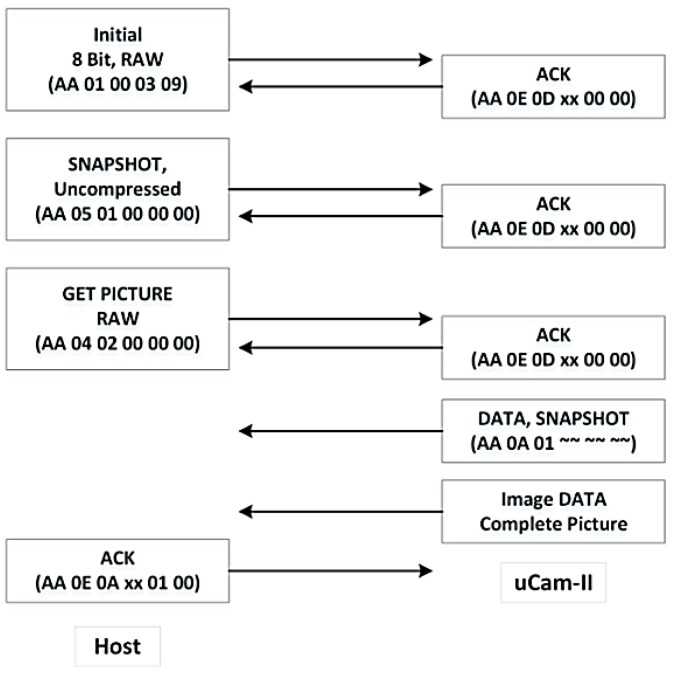
Process of capturing an 8-bit 128 × 128 raw image.

The overall process of capturing an 8-bit grayscale raw image with a resolution of 128 × 128 raw is shown in Figure 5. This resolution is selected because Arduino Due has limited SRAM of 96 KB.

2. Encoding Process

**Figure 6:**
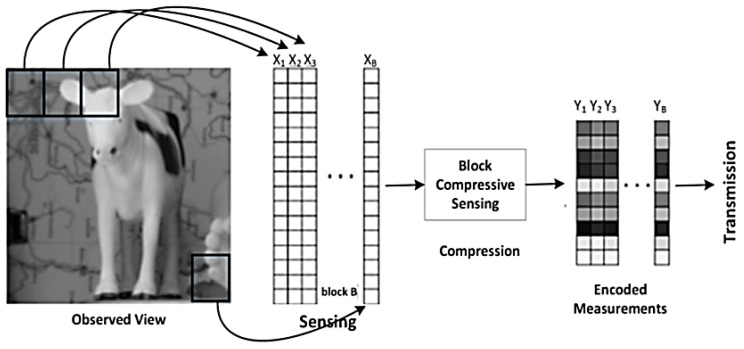
Encoding process of BCS.

The image obtained from uCAM-II is first stored into the flash memory. The BCS is applied to encode the image on a block-by-block basis. The encoding process can be divided into two parts, namely image sensing, and image compression as shown in Figure 6.

In the first part, the raw image of resolution 128 × 128 is first divided into small 16 × 16 independent blocks, and each block is rearranged into a vector with 256-pixel values. This produces a matrix of size 256 × 64, and this is denoted as the sensed measurement, I. Next, I is sampled by random measurement matrix Φ. The measurement matrix Φ used in the JMD scheme is a constrained structure (block diagonal) matrix that is incoherent with any sparsity basis with a very high prospect. This also reduces the memory required to store the measurements when it is implemented as a dense matrix. The size of the measurement matrix Φ is determined based on the block size and sampling rate. For example, if the block size is 16 × 16 and the sampling rate is 0.2, then the Φ generated is of size 51 × 256. Then Φ is multiplied with I to obtain the encoded measurement matrix Y. All the encoded measurement will then be transmitted to the server via the XBee module. However, before transmission, the encoded measurements are quantize using uniform quantization. Each measurement value is converted to a signed 16-bit binary vector. From our analysis, the measurement value can exceed the range of −128 to +128. Hence, it is not sufficient to fit the value into a signed 8-bit binary vector.

3. Wireless Communication

**Figure 7:**
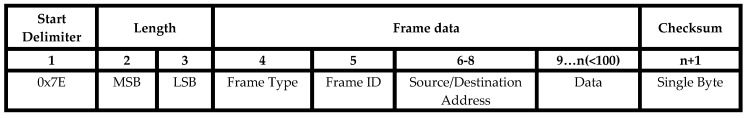
API frame structures for Xbee transmit and receive a request.

Two Series-2 XBee modules are used. One is connected to the Arduino Due, and the other is connected to the server. The former is configured as the end device that is in charge of sending data, whereas the latter is configured as a coordinator that is in charge of setting up the network and receiving data. It is also necessary to ensure that they are operating under the same PAN ID and channel number. All these parameters have to be configured before forming a wireless network. The API mode is used over AT mode to emulate the transmission pattern of a VSN. The API mode is designed to transmit highly structured data in a fast, predictable, and reliable way. The XBee modules were configured in API mode, having a baud rate of 125,000, data bits of 8, no parity bits, and 1 stop bit. In API mode, the input data will be packetized into many API frames before transmit within the wireless network. The API frame structure is shown in Figure 7 [37].

In every API frame, the first byte is a start delimiter that is used to indicate the beginning of each API frame. The value is always 0 × 7E allowing easy detection of a new incoming frame. The next field indicates the length of the frame. The length is of 16 bits value and is divided into MSB (most significant bits) and LSB (least significant bits). After the length is the frame type, frame ID, source or destination address and the payload (data). The frame type indicates how the information is organized in the data field. The frame ID is used to enable a form of acknowledgment that shows the result of the transmission. The source or destination address is a 64-bit value that means either the source or the destination of the packet. The data field contains the information to be transmitted and is dependent on the frame type. 

The value in each field may vary according to transmit or receive the request. For transmit request the frame type, frame ID, 64-bits source or destination address values are 0 × 10, 0 × 01, 0 × 000000000000FFFF (destination address) respectively whereas, for receive request the values are 0 × 91, 0 × 00, 0 × FFFFFFFFFFFFFFFF (source address) respectively. The last field of the API frame is the checksum that is used to test the data integrity. The checksum is calculated by first adding all the bytes in the frame excluding the start delimiter and length, then subtract the lowest 8 bits of the result from 0 × FF.

• **At the Coordinator End:**

**Figure 8:**
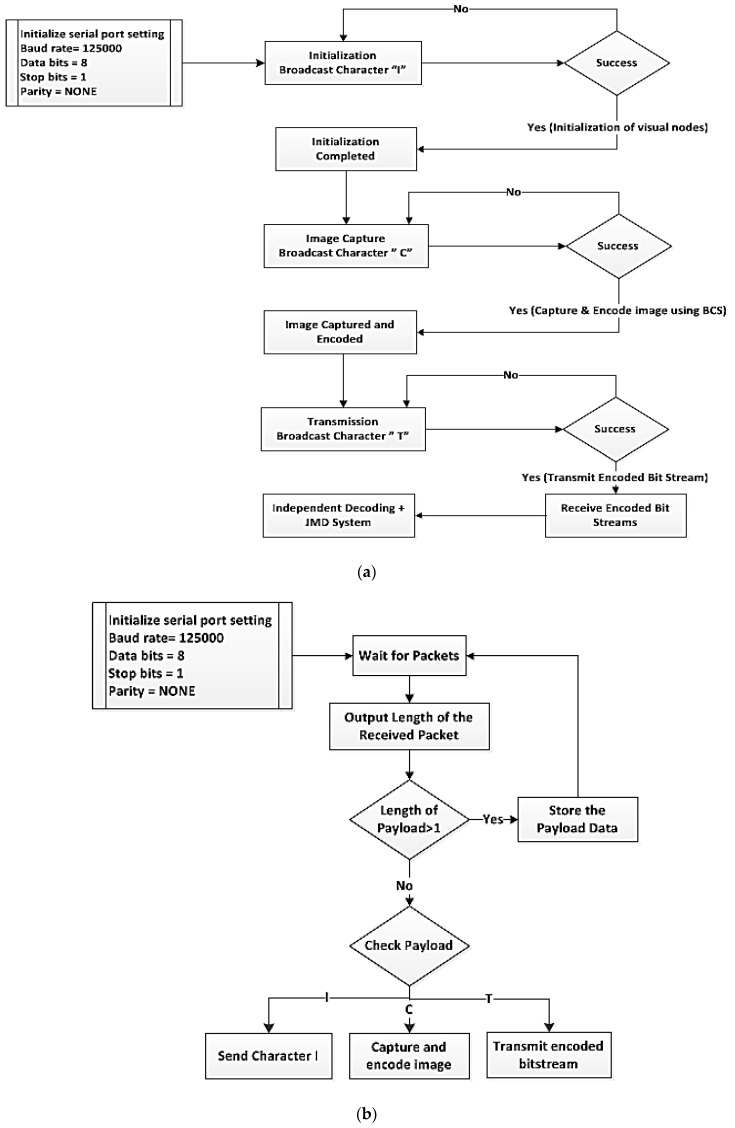
(**a**) Transmission process at the server (coordinator); (**b**) Transmission process at the visual node (end device).

The server is connected to the XBee module (the coordinator) for receiving data transmitted from the visual nodes. Once the coordinator has set up the network, other end devices (visual nodes) will be able to join the network automatically. The communication between the coordinator and the visual node is illustrated in Figure 8a.

○ Initially, the server will broadcast a packet containing an ‘I’ (Initialization) character via the coordinator to all the visual nodes. This step is to define the number of visual nodes in the network (i.e., to identify the number of images that are to be received). If the end device successfully receives the packet, then a ‘Yes’ signal is generated, and an acknowledgment is sent back to the coordinate. If the acknowledgment is not received by the coordinator from the end device for some time, the packet is unsuccessfully and is resent. This initialization step helps to determine the number of visual nodes in the network and to know the number of images that are going to be received. This is followed by broadcasting two more packets containing character C (capturing) and T (transmission) in their respective order.
○ Once the initialization is completed, the coordinator will broadcast the next signal containing a ‘C’ character. The ‘C’ character will update the visual nodes to capture and encode the image data with the BCS scheme.
○ Similarly, after receiving an acknowledgment from the end device, another signal comprising of ‘T’ character is sent to each visual node in the network. As soon as the visual node receives the ‘T’ character, it will start to send the encoded stream to the coordinator (server).
○ After the server has received the encoded stream, the stream will be decoded to recover the captured images by using independent BCS with JMD scheme. As multiple images (due to more than one visual node) will be received at the coordinator (server) end, it is essential to separate the data transmitted by the different visual node. It is done by referring to the automatically embedded source address in the transmitted packet.
○ Finally, the process described above is repeated for the next transmission cycle.

• **At the End Device:**

The XBee module is connected to the Arduino board through a serial port to serve as an end device (visual node). The end device will automatically connect to the initially established network by the coordinator. The communication between the end devices (visual node) and the coordinator is illustrated in Figure 8b.

○ The visual node is always looking for signal (Packet) transmitted from the coordinator (server).
○ Once a packet (API frame) is successfully received, the visual node will process the information acquired from the packet. If the received packet contains an I; the same packet will be transmitted back to the server for acknowledgment purpose. It is from the initialization as discussed in the above section.
○ If the received packet contains ‘C’, the node will capture and encode the images using BCS. The reason for doing this is to synchronize the image capturing process of different visual nodes. This is to ensure that the images are captured at approximately the same time to ensure maximum correlation. Furthermore, this also allows the server to control when the capturing should take place.
○ Once a packet that contains a ‘T’ is received, the visual node will wait for packets (encoded measurements) to packetize the encoded measurements into numbers of API frame, and each frame has a payload size of 72 bytes. All the data will be continuously transmitted to the server until there is no more data to transfer. Then, a packet that carries a value of zero is sent. The purpose of this frame is to inform the server that the previous packet was the end.

#### 3.1.3. Theoretical Basics of Compressive Sensing

CS states that a signal that is sparse in some transform domain could be entirely reconstructed with several samples lower than the requirement stated in Shannon–Nyquist theorem. CS relies on two essential concepts, known as sparsity (signal of interest) and incoherence (sensing modality). 

1. CS Signal Acquisition/Sensing

The signal acquisition process of CS is different from the conventional sensing process. The conventional process operates by collecting a large amount of information and then discards the unnecessary information using compression. CS serves by collecting only the necessary information related to the object of interest by taking certain random projection that is enough for the reconstruction of a signal.

Consider a signal x with length N to be recovered from M measurements (M ≪ N) that is sparse in some transformation domain Ψ with random measurement matrix Φ. The set of measurements y is given as

y = Φ x(1)

where, *x* ∈ R^N^, is the input signal; y ∈ R^M^ is the measurement vector. It is assumed that the random sensing matrix Φ is orthonormal, i.e., Φ Φ^T^ = I. Where I is the identity matrix, M is the number of CS measurements, N = B × B (B=block size) and the sub-rate S is defined as M_B_/N.

2. Reconstruction 

The recovery of the encoded measurements is the main challenge of using CS. As the number of unknowns is much larger than the number of observations, recovery of *x* ∈ R^N^ from its corresponding y ∈ R^M^, i.e., inverse projection of $\hat{x}$ = Φ^−1^ y is ill-posed [16]. Since the signal to be compressed by CS should be sparse in nature, the reconstruction can be carried out by solving a convex optimization problem using sparsity in the transformed domain with either ℓ-norm or image gradient with total variation (TV) norm. 

The reconstruction of a signal *x* lies within the set of sparse significant transformation coefficients *x* = Ψ S and can be obtained by solving the different ℓ-norm optimization problem. The primaryℓ_0_ optimization problem function can be expressed as

(2) $$\hat{x}={\mathrm{argmin}}_{S}{\left\|S\right\|}_{\mathrm{lo}},\;\;\;\;s.t.\;y=\Phi \;\Psi \;S=\Theta S$$

However, solving the ℓ_0_ constrained optimization problem is computationally infeasible due to its combinational and non-differentiable (presence of the absolute value function) property, i.e., nondeterministic polynomial (NP) completeness [5].

Several alternative optimization schemes—such as convex relaxation, greedy-iterative, gradient-descent, and iterative-thresholding—have been proposed to solve Equation (2). However, most of the proposed schemes are exposed to certain issues, such that as the size of the natural image increases, so does the size of the sampling matrix, resulting in higher computational and memory consumption

3. CS-Based Compression Schemes

Generally, CS-based compression schemes can be categorized into full coding and block coding. The former acquires the CS measurements of the visual data by sampling it with appropriate sensing matrix Φ. However, in most cases, Φ is not directly applied to the visual data; rather a sparse transformation is used initially. The Φ is then applied to transform coefficients to attain the CS measurements. 

In contrast, the latter acquires the CS measurements by first dividing the visual data into the small independent block. Each block is then individually sampled by the same sensing matrix Φ. Such an approach helps to reduce the computational complexity and memory requirements at the encoder and is appropriate for low power applications such as VSN. 

In [16], a block-coding-based CS scheme is proposed. The scheme denoted as block-based compressive sensing (BCS) attempts to process an image on a block-by-block basis. An image is first divided into small BxB independent block. Each block is then individually sampled using the same measurement matrix Φ with a constrained (block-diagonal) structure as shown in Equation (3).

(3) $$\Phi =\left[\begin{array}{ccc} \phi_{B} & \cdots & 0\\ \vdots & \ddots & \vdots \\ 0 & \cdots & \phi_{B} \end{array}\right]$$

The benefits of using BCS include:

(i) The implementation and storage of the measurement operator are simple;
(ii) Block-based measurement is more expedient for practical applications;
(iii) The individual processing of each block of image data results in the easy initial solution.

The two basic variants that can be used to reconstruct measurements encoded using BCS, known as smooth projected Landweber (SPL) and total variation (TV) minimization. However, in our research, we have used the joint multiphase decoding (JMD) framework for the reconstruction of images that make use of the TV minimization approach and is referred to as BCS-JMD-TV. A brief overview of the scheme is described as follows, the details of the scheme can be referred from [17,18] 

4. BCS-JMD-TV

BCS-JMD-TV [17,18] is a multi-view compression scheme for VSN based on block-based compressive sensing (BCS) and joint multi-phase decoding (JMD). First, images captured by different visual nodes are encoded using BCS to reduce the hardware complexity. The block-based approach simplifies the implementation and storage of the visual node and provides significantly faster reconstruction. One of the visual nodes is configured to serve as the reference node, whereas the others serve as non-reference nodes. In this case, images captured by the non-reference nodes are encoded at a lower subrate when compared with the images from the reference nodes. The core idea is to improve the reconstruction of images captured by the non-reference nodes, by using information in the image captured by the reference node. It is achieved by exploiting the high correlation between them at the joint-decoder. The encoded measurements are then transmitted independently to the server that serves at the joint-decoder. 

**Figure 9:**
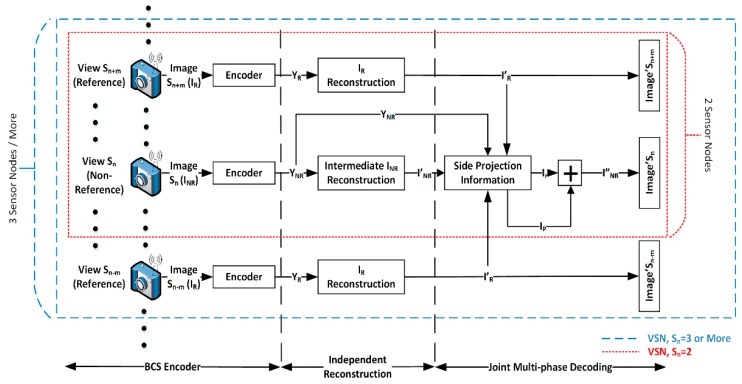
Overview of the joint multiphase decoding (JMD) scheme.

At the joint-decoder, the JMD is applied to the received images. The JMD produces and uses side projection information (SPI) to aid the reconstruction of the final image. One reason for using BCS is that it managed to provide an initial reconstruction of an image in a shorter period [16]. The initial reconstruction helps in the generation of the SPI, which is the core component of the scheme. Besides using the initial reconstruction, residual reconstruction and prediction methods are added to produce an SPI that could better represent the visual data to be decoded. The scheme also works well for both near-field and far-field images, and could also handle parallax and occlusion issues. It is achieved by aligning and fusing the images captured from different view angles. Furthermore, the JMD relies on simplified operations that are less complex when compared to the other reconstruction schemes. Experimental results presented in [17] show that the BCS-JMD scheme can be applied to images with low, medium, and high texture variations. It can outperform the different independent BCS compressions by a margin of 1.5 dB to 3 dB at various subrates. Furthermore, when compared with other standard multi-view CS compression schemes, the proposed scheme shows a gain of 1.5–2 dB at lower subrates, and the reconstruction speed is also 30–40% shorter. The complete JMD framework is shown in Figure 9.

## 4. Experimental Results

### 4.1. Experimental Setup

**Figure 10:**
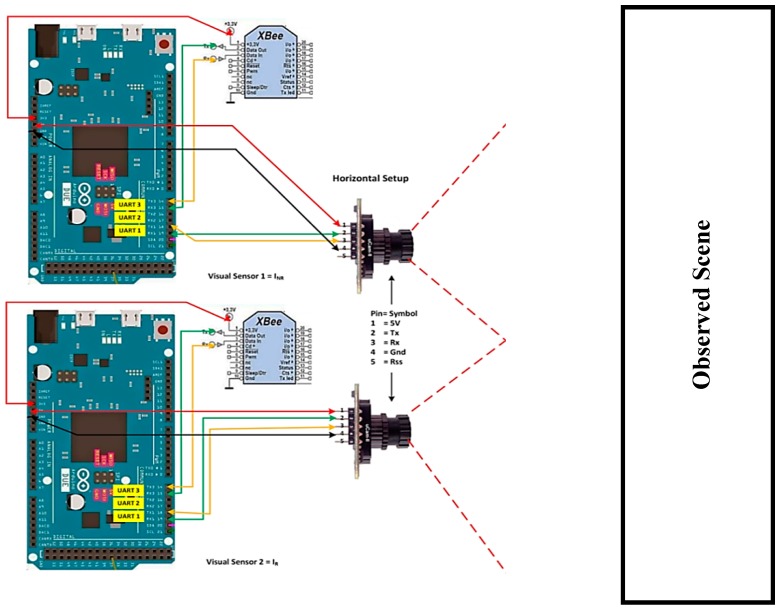
Proposed multi-visual setup.

In order to simplify the evaluation process, two visual nodes are deployed in a horizontal setup, and each visual sensor is separated by a specific distance from its neighbor as shown in Figure 10. 

However, the setup can be extended by adding more visual nodes. In this case, the BCS compression scheme as described in Section 3.1.3 is implemented. Hence, one of the visual nodes is configured as the non-reference node and the other as the reference node. The images captured are of 8-bit grayscale format with a resolution of 128 × 128. All the images are encoded independently using the BCS. Images captured by the non-reference node are encoded at lower subrates range from 0.05 to 0.3. The idea is to improve the images captured by the non-reference node with the help of images captured by the reference node. The encoded measurements from the two visual nodes are then transmitted using the XBee module to the server for reconstruction.

**Figure 11:**
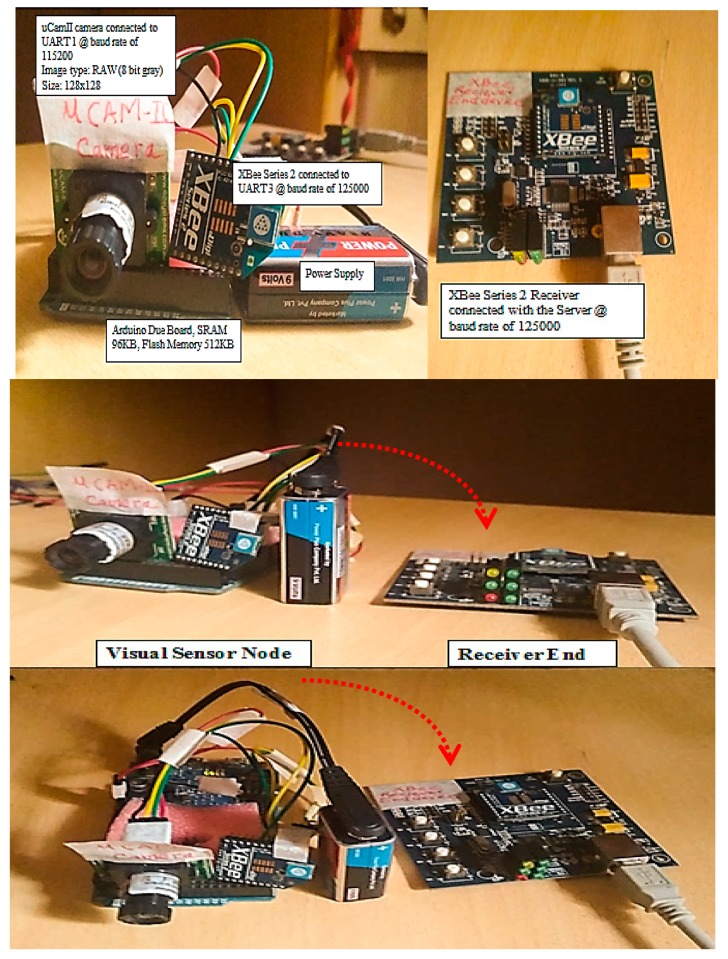
Different views of the constructed visual node and its transmission setup.

The server is equipped with an Intel(R) Xeon(R) E5-1620 CPU running at 3.6 GHz and 8 GB of RAM with Windows operating system. The server is used to reconstruct the encoded measurements by using the JMD. It is implemented using MATLAB ver. 8.3.0.532 (R2014a). Because the server will be receiving images from different visual nodes, it is important to differentiate the origin of the data. In order to achieve this, the server will refer to the source address embedded in the received packet. Figure 11 shows different views of the constructed visual node platform and its transmission setup.

The evaluation is carried out by measuring the execution time and energy consumption for capturing, encoding, and transmission of visual data in seconds (s) and joules (J) respectively at various sampling rates. Moreover, to validate the effectiveness of the reconstruction scheme in terms of visual quality, the peak signal-to-noise ratio (PSNR (dB)) and structural similarity index metric (SSIM) are also measured. All the images captured by the non-reference node are encoded at lower subrates of 0.05, 0.1, 0.15, 0.2, 0.25, and 0.3, whereas images captured by the reference node are encoded at a fixed subrate of 0.3. In addition to this, the effect of using block size 8 × 8 and 16 × 16 for BCS is also compared.

#### 4.1.1. Execution and Transmission Time Analysis

The total time required to perform the capturing, encoding, and transmission of visual data is presented in Table 1. The image capturing time and sensing time for both block size is about the same. It is noted that the image encoded with block size 8 × 8 is 3–4 times faster in terms of execution time than block size 16 × 16. It is due to the extra bytes produced by using a larger block size. Subsequently, an image encoded with a block size of 8 × 8 takes 6.72–12.24% less transmission time than a block size of 16 × 16.

#### 4.1.2. Energy Consumption Analysis

The energy consumption at different stages is measured by taking the product of measured power and measured time (Energy = Power * Time). The time required is already measured in Table 1. The power is assessed by measuring the current drain at each stage independently, whereas the voltage remains constant at 3.3 V.

The results obtained are shown in Table 2. The stages include standby, capturing, encoding, and transmission. In the standby stage, the visual nodes are waiting for instructions from the server. The capturing stage refers to the capturing of an image. The encoding stage is the sensing and compression of the captured image. Finally, the transmission stage refers to the transmission of encoded bits stream from visual node to the workstation. All the measurement was done by using the Unity True RMS Multi-meter. All the power values are presented in Watts (W).

The results show that the power required for encoding of an image is 0.05 watt that is 52.2–62.4% less than the power needed for the transmission of the encoded bitstream, which is 0.122 watts. Moreover, the power consumption during standby is 0.35 W, and the total power consumption with encoding and transmission is 0.52 W.

Table 3 presents the energy consumed at different stages using block size 8 × 8 and 16 × 16 for various subrates. The results show that the energy consumed during encoding when using block size 8 × 8 is 2–3 times less than block size 16 × 16. Subsequently, the transmission also consumed 8.4–13.4% less energy.

The energy required for encoding is 40–60% less than the energy required for transmission. It validates the statement in [38] that transmission of data requires more energy when compared to processing. It should also be noted that the energy consumed by the visual node when in standby is 1.43 J, which is a bit on the higher side. However, the value presented is not the exact appearance of energy consumption as we did not enable sleep mode or reduce the clock frequency. It is expected that the idle state consumption can be further reduced to a greater extent by applying all these power management strategies.

#### 4.1.3. Visual Quality Analysis

**Figure 12:**
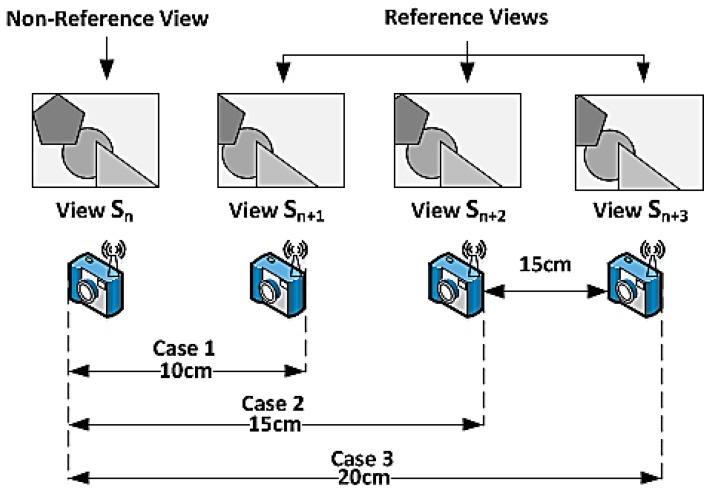
Different separation setups.

The visual nodes are placed horizontally aligned side by side, and each visual node is separated by a specific distance from its neighbor as shown in Figure 12. One of the visual nodes is configured as the non-reference node, and the other is configured as the reference node. Furthermore, the observed scenes are shown in Table 4.

The results of comparing the BCS-JMD-TV with independent BCS (BCS-TV-AL3) are presented in Table 5. For smaller separations (10 cm) the JMD-TV provides an average gain of 1.5 dB to 2.5 dB, whereas for larger separation (20 cm) the gain reduces to an average of 1 dB to 2 dB when moving from higher to lower subrates. Generally, the JMD-TV scheme produces poor results if the camera separation is too large. As the distance separation increases, the correlation between them decreases, leading to less accurate registration and fusion of the images. Furthermore, it is also noticed that larger block size generates 0.5 dB to 0.8 db better reconstruction than the smaller block size.

**Figure 13:**
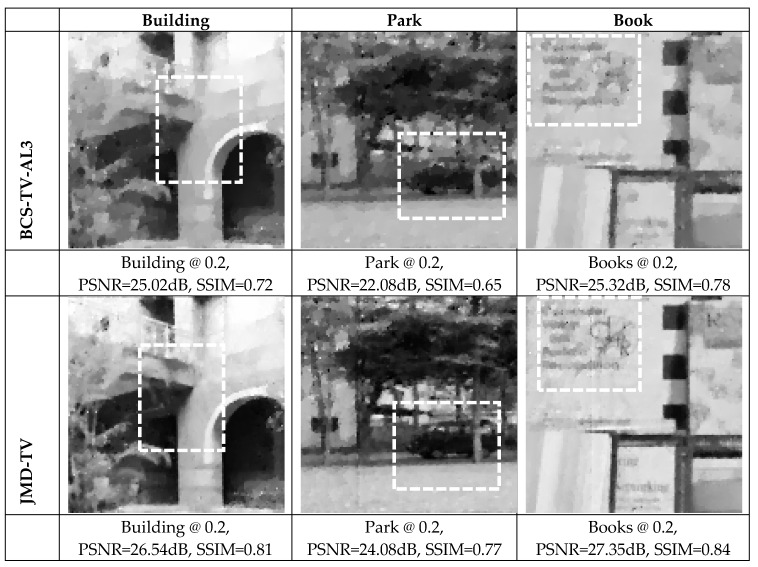
Visual quality comparison of images encoded using independent BCS-TV-AL3 and JMD-TV at subrates 0.2, block size 16 × 16, and visual nodes separation of 15 cm.

Some samples of the reconstructed images are shown in Figure 13. By comparing the highlighted regions (white dotted boxes), it is noticed that the JMD-TV reduces the blurring effect present in the images reconstructed using BSC-TV-AL3, and the reconstructed image looks much sharper.

#### 4.1.4. Complexity and Energy Consumption Comparison

The computational complexity and energy consumption of using BCS with different block sizes are compared with the case no compression (raw) and the case of using JPEG compression (JPEG). In each case, the time and energy taken to encode and transmit an image are measured. When BCS is applied to encode the images, block sizes (B) of 8 × 8 and 16 × 16 are evaluated. In both situations, a subrate (M) of 0.3 is used.

The results in Table 6 show that the transmission of the image without compression requires more time and energy. It can be noted that JPEG compression consumes 30% less energy and BCS consumes 50–60% less energy than the case of the uncompressed raw image. At a subrate of 0.3, BCS with block sizes of 8 × 8 and 16 × 16 require 60% and 10% less encoding time respectively when compared to JPEG compression. In terms of transmission time and energy consumption, the BCS outperforms JPEG by a margin of 30–40%.

## 5. Conclusions

A visual node prototype has been developed to evaluate the performance of BCS in terms of computational complexity and reconstruction quality in a real-world scenario. BCS is implemented on the visual node to encode the captured image before transmission, and JMD framework was applied on the encoded image to improve the reconstruction quality. The evaluations show that the energy taken to transmit an image is 50% higher than that of compressing the image. Hence, it is wise to compress the image before transmission takes place. When compared with the case of no compression and when JPEG is used to compress the captured image, the total energy consumption (encoding + transmission) is 40–60% lower when block size of 8 × 8 is used, whereas for a block size of 16 × 16 the energy consumed by the proposed scheme is 10–20% lower. Furthermore, the JMD-TV shows promising reconstruction quality as compared to conventional BCS-TV.

## Figures and Tables

**Table 1:** Time taken to complete the encoding and transmission at various block sizes and subrates.

Sub Rate	Size in Bytes	Image Capture Time (s)	Total Encoding Time (s)		Total Transmission Time (s)	Total Encoding + Transmission Time (s)
			Sensing Time	Compression Time		
**Block Size = 8**						
0.05	1183	1.41	0.011	0.111	0.460	0.582
0.10	2233	1.41	0.011	0.222	0.965	1.198
0.15	3913	1.41	0.011	0.370	1.575	2.056
0.20	5010	1.41	0.011	0.481	2.167	2.659
0.25	6243	1.41	0.011	0.592	2.649	3.252
0.30	7273	1.41	0.011	0.702	3.167	3.887
**Block Size = 16**						
0.05	1288	1.41	0.011	0.478	0.524	1.013
0.10	2653	1.41	0.011	0.957	1.107	2.075
0.15	4276	1.41	0.011	1.398	1.661	3.070
0.20	5423	1.41	0.011	1.877	2.244	4.132
0.25	6343	1.41	0.011	2.356	2.805	5.172
0.30	7875	1.41	0.011	2.834	3.383	6.228

**Table 2:** Power consumed at different stages.

Operating Stages	Voltage (V)	Total Current (mA)	Average Current (mA)	Average Power V × I (W)
Standby	3.3	107.5–108.2	107.80	0.350
Image Capture	5.0	80.17–85.10	82.63	0.410
Encoding	3.3	15.50–15.90	15.70	0.0521
Transmission	3.3	37.7–37.9	37.8	0.1221
Standby + Encoding	3.3	122.9–124.1	123	0.407
Standby + Encoding + Transmission	3.3	159.8–160.4	160.1	0.528

**Table 3:** Energy consumption using block size 8 × 8 and 16 × 16 at various subrates.

Sub Rate	Idle State (J)	Image Capture (J)	Encoding (J)	Transmission (J)	Total Encoding + Transmission (J)
**Block Size 8**					
0.05	1.43	0.58	0.006	0.056	0.062
0.10	1.43	0.58	0.012	0.118	0.130
0.15	1.43	0.58	0.019	0.205	0.224
0.20	1.43	0.58	0.025	0.265	0.290
0.25	1.43	0.58	0.031	0.323	0.354
0.30	1.43	0.58	0.036	0.386	0.424
**Block Size 16**					
0.05	1.43	0.58	0.025	0.064	0.089
0.10	1.43	0.58	0.050	0.135	0.185
0.15	1.43	0.58	0.072	0.203	0.275
0.20	1.43	0.58	0.097	0.274	0.371
0.25	1.43	0.58	0.122	0.342	0.464
0.30	1.43	0.58	0.147	0.418	0.560

**Table 4:** Sample images captured by the visual nodes.

	**Non-Reference Image**	**Reference Image @ 10 cm Separation**	**Reference Image @ 15 cm Separation**	**Reference Image @ 20 cm Separation**
**Building**	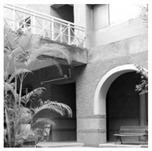	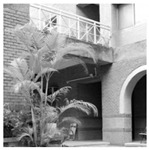	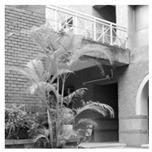	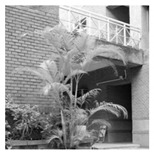
**Books**	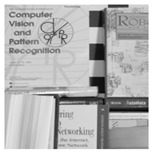	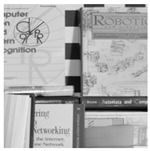	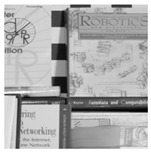	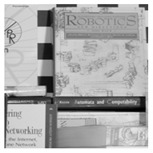
**Park**	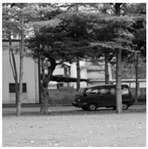	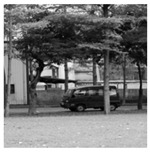	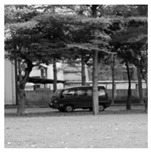	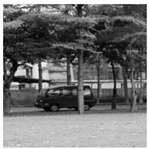

**Table 5:** Performance (PSNR (dB)) comparison of using the JMD compression scheme with different camera separations, block sizes, and subrates.

	**Building**							
	**Reference Views**	**Subrate**	**0.05**	**0.1**	**0.15**	**0.2**	**0.25**	**0.3**
Block Size 8		BCS-TV-AL3	19.93	21.23	23.30	24.85	25.42	26.43
	S_n±1_ = 10 cm	JMD-TV	**22.34**	**23.56**	**25.56**	**26.95**	**27.47**	**28.32**
	S_n±2_ = 15 cm	JMD-TV	21.75	22.95	24.98	26.41	26.89	27.74
	S_n±3_ = 20 cm	JMD-TV	21.18	22.45	24.41	25.79	26.28	27.22
Block Size 16		BCS-TV-AL3	21.24	22.97	23.99	25.02	26.02	26.69
	S_n±1_ = 10 cm	JMD-TV	**23.76**	**25.27**	**26.11**	**27.12**	**27.99**	**28.52**
	S_n±2_ = 15 cm	JMD-TV	23.12	24.70	25.65	26.54	27.45	27.98
	S_n±3_ = 20 cm	JMD-TV	22.64	24.20	25.08	25.95	26.91	27.44
	**Park**							
	**Reference Views**	**Subrate**	**0.05**	**0.1**	**0.15**	**0.2**	**0.25**	**0.3**
Block Size 8		BCS-TV-AL3	17.84	19.49	20.66	22.13	22.76	23.92
	S_n±1_ =10 cm	JMD-TV	**20.67**	**22.24**	**23.27**	**24.43**	**25.00**	**25.98**
	S_n±2_ = 15 cm	JMD-TV	20.12	21.67	22.75	23.97	24.51	25.47
	S_n±3_ = 20 cm	JMD-TV	19.71	21.11	22.21	23.52	23.97	24.93
Block Size 16		BCS-TV-AL3	18.59	20.12	21.04	22.07	22.99	24.02
	S_n±1_ = 10 cm	JMD-TV	**21.75**	**23.27**	**23.78**	**24.68**	**25.5**	**26.31**
	S_n±2_ = 15 cm	JMD-TV	21.22	22.70	23.17	24.05	24.95	25.83
	S_n±3_ = 20 cm	JMD-TV	20.66	22.12	22.63	23.54	24.44	25.31
	**Book**							
	**Reference Views**	**Subrate**	**0.05**	**0.1**	**0.15**	**0.2**	**0.25**	**0.3**
Block Size 8		BCS-TV-AL3	18.41	20.99	22.84	24.75	25.61	26.54
	S_n±1_ = 10 cm	JMD-TV	**21.09**	**23.73**	**25.44**	**27.07**	**27.87**	**28.50**
	S_n±2_ = 15 cm	JMD-TV	20.82	23.36	25.14	26.89	27.56	28.22
	S_n±3_ = 20 cm	JMD-TV	20.15	22.67	24.45	26.33	27.05	27.88
Block Size 16		BCS-TV-AL3	19.58	22.31	23.72	25.32	26.66	27.66
	S_n±1_ = 10 cm	JMD-TV	**22.53**	**25.20**	**26.38**	**27.81**	**28.78**	**29.61**
	S_n±2_ = 15 cm	JMD-TV	22.25	24.88	26.09	27.35	28.55	29.33
	S_n±3_ = 20 cm	JMD-TV	21.98	24.49	25.78	26.98	28.17	29.10

Note: Bold values relate to the maximum PSNR (dB) reached for a given subrate and image.

**Table 6:** Comparison of computational complexity and energy consumption with and without using compression.

Size of Raw Image = 128 × 128						
Image Type	Encoding Time (s)	Encoding Power (W)	Encoding Energy (J)	Transmission Time (s)	Transmission Power (W)	Transmission Energy (J)
Raw	-	-	-	8.20	0.122	1.004
JPEG	3.015	0.052	0.156	6.39	0.122	0.781
BCS B = 8 × 8 M = 0.3	0.713	0.052	0.037	3.16	0.122	0.385
BCS B = 16 × 16 M = 0.3	2.845	0.052	0.105	3.38	0.122	0.412
